# Influence of Consumption of a High-Protein vs. High-Carbohydrate Meal on the Physiological Cortisol and Psychological Mood Response in Men and Women

**DOI:** 10.1371/journal.pone.0016826

**Published:** 2011-02-03

**Authors:** Sofie G. Lemmens, Jurriaan M. Born, Eveline A. Martens, Mieke J. Martens, Margriet S. Westerterp-Plantenga

**Affiliations:** 1 Top Institute Food and Nutrition, Wageningen, The Netherlands; 2 Department of Human Biology, Maastricht University, Maastricht, The Netherlands; Paris Institute of Technology for Life, Food and Environmental Sciences, France

## Abstract

Consumption of meals with different macronutrient contents, especially high in carbohydrates, may influence the stress-induced physiological and psychological response. The objective of this study was to investigate effects of consumption of a high-protein vs. high-carbohydrate meal on the physiological cortisol response and psychological mood response. Subjects (n = 38, 19m/19f, age = 25±9 yrs, BMI = 25.0±3.3 kg/m^2^) came to the university four times, fasted, for either condition: rest-protein, stress-protein, rest-carbohydrate, stress-carbohydrate (randomized cross-over design). Stress was induced by means of a psychological computer-test. The test-meal was either a high-protein meal (En% P/C/F 65/5/30) or a high-carbohydrate meal (En% P/C/F 6/64/30), both meals were matched for energy density (4 kJ/g) and daily energy requirements (30%). Per test-session salivary cortisol levels, appetite profile, mood state and level of anxiety were measured. High hunger, low satiety (81±16, 12±15 mmVAS) confirmed the fasted state. The stress condition was confirmed by increased feelings of depression, tension, anger, anxiety (AUC stress vs. rest p<0.02). Consumption of the high-protein vs. high-carbohydrate meal did not affect feelings of depression, tension, anger, anxiety. Cortisol levels did not differ between the four test-sessions in men and women (AUC nmol·min/L p>0.1). Consumption of the test-meals increased cortisol levels in men in all conditions (p<0.01), and in women in the rest-protein and stress-protein condition (p<0.03). Men showed higher cortisol levels than women (AUC nmol·min/L p<0.0001). Consumption of meals with different macronutrient contents, i.e. high-protein vs. high-carbohydrate, does not influence the physiological and psychological response differentially. Men show a higher meal-induced salivary cortisol response compared with women.

## Introduction

Recent human studies have shown a possible relationship between stress and the increased prevalence of obesity [1], [2], [3], [4]. The stress response involves the hypothalamus pituitary adrenal (HPA) axis, which regulates the secretion of its end-product cortisol [3]. Chronic stress is associated with hyperactivity of the HPA axis and consequently increased cortisol levels, which have been associated with visceral fat accumulation and obesity [1], [5], [6]. During stress, food choice is often shifted towards sweet and fat foods, possibly because they are perceived as highly rewarding [7], [8], [9]. However, consumption of some of these preferred or highly rewarding foods, namely carbohydrates, may not reduce stress but even increase stress, i.e. increased HPA-axis activity, represented by cortisol concentrations. A study by Vicennati et al. [10] showed that, in contrast to a high-protein/fat meal, a high-carbohydrate meal significantly increased the plasma cortisol levels in visceral obese subjects. Lacroix et al. [11] showed that high-protein/high-fat foods reduce cortisol concentrations remarkably in rats. Moreover, a study by Martens et al. [12] investigating the effects of single macronutrients on serum cortisol concentrations in normal weight men showed that the cortisol response to consumption of carbohydrates was higher than the cortisol response to consumption of fats or proteins. Carbohydrates increased serum cortisol concentrations while fat as well as protein did not relative to water [12].

On the other hand, Gibson et al. [13] and Slag et al. [14] showed increased cortisol levels induced by a protein-rich meal. A study by Gonzalez-Bono et al. [15] showed neither a difference between the effects of macronutrients on salivary cortisol levels, nor a cortisol response to meal consumption. Lovallo et al. [16] showed no meal-induced salivary cortisol response in the case of a mental stressor followed by a meal but did show a meal-induced cortisol response in the case of a physical stressor followed by a meal.

These studies show that the effects of macronutrients on the response of the HPA axis are still controversial. Little is known about the response of a physiological challenge such as food intake following a psychological stress challenge. This study was, therefore, carried out to investigate possible effects of consumption of comparable meals with different macronutrient contents (high-protein vs. high-carbohydrate) on the physiological cortisol response under stress. Moreover, we wanted to investigate the possible effects of high-protein vs. high-carbohydrate meals on the psychological mood response. Increases in negative mood in response to stressors can lead to greater food intake [9], [17]. Consumption of foods that improve the stress-induced mood state may prevent further intake of energy-dense foods. Studies by Markus et al. [18], [19] showed that carbohydrate-rich, protein-poor foods improve mood and stress coping following acute stress-inducing tasks, only in stress-vulnerable subjects, possibly due to increased levels of brain tryptophan and serotonin. Firk et al. [20] showed that intake of tryptophan-rich hydrolyzed protein increased positive mood and dampened the cortisol response to acute stress.

We hypothesized that high-protein foods, in contrast to comparable high-carbohydrate foods, would not increase salivary cortisol concentrations more under stress and consequently would improve mood.

## Methods

### Ethics Statement

All procedures were carried out with the adequate understanding and written consent of the subjects. The study was approved by the Medical Ethical Committee of the Maastricht University, and was in compliance with the Declaration of Helsinki. The study was registered in the Dutch Trial Register (NTR, TC = 1904). The protocol described here in this study deviates from the trial protocol approved by the Medical Ethical Committee of the Maastricht University as it comprises only a part of the approved trial protocol.

### Subjects

Thirty-eight Caucasian subjects (19m/19f; age 25±9 yrs (mean±SD, range 18–51 yrs)) with a body mass index (BMI) of 25.0±3.3 kg/m^2^ (mean±SD, range 18.9–30.5 kg/m^2^) participated in this study. Based upon the study by Vicennati et al. [10], power analysis showed that with an α of 0.0125 (taking into account the Bonferroni correction for multiple testing) and β of 0.10 (power = 1-β = 0.90), at least 31 subjects were needed. Subjects were recruited by advertisements in local newspapers and on notice boards at the university. They underwent an initial screening including measurement of body weight, height, waist circumference and hip circumference, and completion of a questionnaire related to health, use of medication, smoking behavior, alcohol consumption, physical activity and eating behavior. Eating behavior was analyzed using a validated Dutch translation of the Three Factor Eating Questionnaire (TFEQ) which measures three components: ‘cognitive restraint of eating’ (factor 1), ‘disinhibition of restraint’ (factor 2), and ‘subjective feeling of hunger’ (factor 3) [21].

### Study design

The study was conducted in a randomized cross-over design. All subjects came to the university four times in a fasted state between 08:00 and 9:00 AM, once for a stress test session receiving a high-protein meal, once for a rest test session receiving a high-protein meal, once for a stress test session receiving a high-carbohydrate meal, and once for a rest test session receiving a high-carbohydrate meal. The order of the four conditions was randomized across the subjects to prevent any order effects.

**Figure 1:**
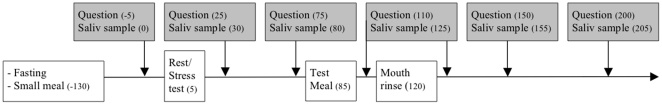
Schematic overview of the study design. Numbers in brackets represent the time points (in min) at which data was collected or tasks were completed. ‘Question’, questionnaires; ‘Saliv sample’, salivary sample.

**Figure 1** gives a schematic overview of the study design. After arrival at the university, subjects were seated in the laboratory and remained seated throughout the experiment. All subjects received 50 g of yoghurt (‘Campina magere yoghurt naturel’, 84 kJ, Energy% Protein/Carbohydrate/Fat (En% P/C/F) 53/44/2) to prevent extreme hunger feelings. The test sessions started two hours later, to overcome the high cortisol morning peak and consequently to prevent the more difficult detection in salivary cortisol changes. Moreover, the two-hour waiting period gave the subjects the chance to adapt to the laboratory environment. During those two hours subjects remained seated and read a book or magazine.

An ego threatening computer test containing elements of an IQ-test was used to create the stress vs. rest conditions in subjects [9], [22], [23]. Two versions of the computer test were used: a difficult stress version with not enough time to solve the assignments and an easier control version with enough time to solve the assignments. This computer test was an updated version of the test used by Rutters et al. [9] and Born et al. [24] and had a duration of 20 min. Subjects were given the computer test before consumption of the test meal. This test meal (lunch) was either a high-protein meal or a high-carbohydrate meal, which had to be consumed entirely within 30 min. After the meal subjects rinsed their mouth thoroughly with cold water, prior to salivary sample collection.

The stress response was determined by means of salivary cortisol concentrations, Profile Of Mood State (POMS) and State Trait Anxiety Inventory (STAI) questionnaires. One hundred unit visual analogue scales (VAS; in mm) were used to assess the appetite profile. Salivary samples and questionnaires were collected six times per test session.

All women were tested in the follicular phase, as it has been shown that women have a higher spontaneous energy intake in the luteal phase compared with the follicular phase [10], [25].

### Test meals

The test meal was either a high-protein lunch (En% P/C/F 65/5/30) or a high-carbohydrate lunch (En% P/C/F 6/64/30). Both meals were comparable and matched for energy density: 4 kJ/g. The amount of the meals that was given to the subjects corresponded to 30% of their daily energy requirements (DER). For each subject the DER were calculated by multiplying the basal metabolic rate (BMR) by the appropriate physical activity factor (1.5–1.8, derived from the screening questionnaire, [26]). The BMR (kcal/day) was calculated according to the equation of Harris–Benedict [27].

The high-protein meal consisted of a salad (iceberg lettuce, cucumber, mushroom, and sunflower oil), Gouda cheese, salami, and a strawberry protein shake. The high-carbohydrate meal consisted of a salad (iceberg lettuce, cucumber, green pepper, and sunflower oil), savory cheese biscuits and TUC bacon biscuits, and a strawberry carbohydrate shake. In both meals the shakes represented 47 En% of the total meal. Beforehand, during screening, subjects had to taste and rate the food items for subjective liking (VAS), in order to check whether all food items were acceptable.

### Questionnaires

One hundred unit VAS (mm) were used to assess the appetite profile. The scales were anchored with ‘not at all’ at one end and ‘extremely’ at the other end, and combined with questions on feelings of hunger, thirst, fullness, satiety, and desire to eat, and on subjective liking and wanting of the test meals.

Mood states were assessed using a modified version of the Dutch translation of the POMS [28]. This questionnaire contains 35 adjectives that are rated on a five-point scale and is divided into five subscales (depression, tension, confusion, fatigue, and anger). The Dutch translation of the state scale of the STAI questionnaire was used to measure state anxiety [29]. Subjects had to rate 20 statements on how they felt at that moment on a four-point scale. An increase in POMS and STAI scores is associated with a worsening in mood.

The VAS, POMS and STAI questionnaires were completed six times throughout the test sessions at −5, 25, 75, 110, 150, and 200 min (Figure 1).

Beforehand, during screening, subjects were familiarized with the questionnaires.

### Saliva samples

To determine salivary cortisol levels, six saliva samples were collected at 0, 30, 80, 125, 155, and 205 min (Figure 1) with the help of cotton swabs (Salivettes, Sarstedt, Etten-Leur, The Netherlands). Subjects were instructed to gently chew on the swab for one min. Cotton swabs were then transferred to the plastic containers and stored at −20°C until analysis. During screening subjects had the chance to chew on a swab in order to get used to the procedure.

Salivary cortisol concentrations were measured by the laboratory of Prof. Dr. C. Kirschbaum, Dresden University of Technology, Germany. After thawing, saliva samples were centrifuged at 3000 rpm for 10 min. Luminescence Immunoassay (IBL, Hamburg, Germany) with intra- and inter-assay precision of 2.5% and 4.7%, respectively, was used to measure salivary cortisol concentrations.

### Statistics

Data were analyzed using StatView 5.0 (SAS Institute Inc., Cary, NC, USA). ANOVA with repeated measures was used to study the conditional effects of stress vs. rest and of high-protein vs. high-carbohydrate, and the effects of time, on cortisol level measurements and questionnaire data (POMS, STAI, VAS). Factorial ANOVA was used to analyze differences between men and women. Paired and unpaired Student's t-tests were used as Post hoc analyses for significant interactions to aid interpretation. Simple linear regression models were used for correlation analysis between parameters. Areas under the curve (AUC) for cortisol and questionnaire data were calculated using the trapezoid method. All tests were two-sided and differences were considered significant at p<0.05. Values are expressed as mean ± standard error of the mean (SEM), unless stated otherwise.

## Results

### Subject characteristics

The characteristics of the subjects are summarized in **Table 1**. No significant differences were shown between men and women in age, BMI, hip circumference, and disinhibition score. Women showed a significantly higher dietary restraint score and feeling of hunger score when compared with men (p<0.05). Men showed significantly higher height, body weight, waist circumference, and salivary cortisol concentrations (AUC) when compared with women (p<0.05). Therefore, the results of men and women were analyzed separately.

**Table 1:** Characteristics of men and women.

	Men (n = 19)	Women (n = 19)	p^a^
Age (y)	25.6±8.6	24.9±9.3	n.s.
Height (cm)	180.2±7.7	168.6±6.4	<.0001
Body weight (kg)	80.1±8.8	71.6±9.4	<.01
BMI (kg/m^2^)	24.8±3.4	25.2±3.2	n.s.
Waist circumference (cm)	86.4±9.7	79.9±9.9	<.05
Hip circumference (cm)	103.7±5.5	105.5±5.1	n.s.
Dietary restraint score	4.7±3.7	7.5±4.0	<.05
Disinhibition score	3.9±1.4	5.1±2.9	n.s.
Feeling of hunger score	3.1±2.3	5.6±3.4	<.01

Values are means±SD.

a p-value: differences between men and women (factorial ANOVA).

n.s. = non-significant.

### Stress parameters

**Figure 2:**
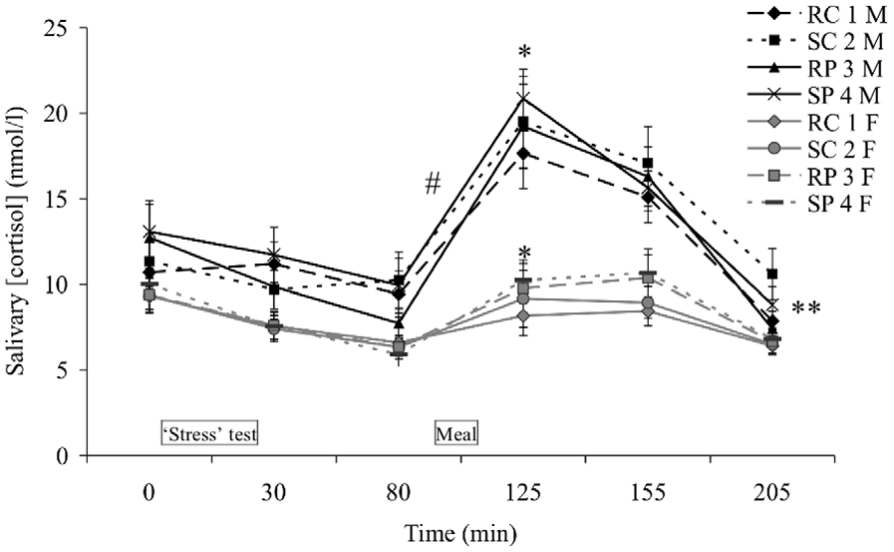
Salivary cortisol concentrations (mean±SEM) at six time points (0, 30, 80, 125, 155, and 205 min) throughout the four test sessions: rest-carbohydrate (RC), stress-carbohydrate (SC), rest-protein (RP), stress-protein (SP); for men (n = 19, M) and women (n = 19, F). **p<0.0001 for overall (AUC) higher cortisol levels in men vs. women; #p<0.05 for higher meal-induced increase in cortisol levels in men vs. women (time point 80–125 min); *p<0.03 for increased cortisol levels in men in all conditions, in women in RP and SP (time point 80 vs. 125 min).

Salivary cortisol levels were analyzed for men and women separately. Salivary cortisol levels did not differ between the conditions of stress vs. rest and high-protein vs. high-carbohydrate in men and women (AUC and per time point, **Figure 2**). There was an overall effect of time on salivary cortisol levels in men and women (p<0.0001). Consumption of the test meals (time point 80–125 min, Figure 2) induced increased salivary cortisol levels in men in all conditions (p<0.01) and in women in the rest-protein and stress-protein condition (p<0.03). This meal-induced increase in cortisol levels was higher in men compared with women in all conditions (p<0.05). Men showed overall higher salivary cortisol levels compared with women (AUC p<0.0001; Figure 2), in all conditions. Cortisol baseline values (time point 0 min, Figure 2) did not differ between men and women, in all conditions.

Men had a higher waist-to-hip ratio compared with women (p<0.01) and simple regression analysis showed a positive relationship between cortisol levels (AUC) and waist-to-hip ratio (p<0.04, R^2^ = 0.1).

POMS and STAI questionnaires showed higher feelings of depression, tension, anger, and anxiety during the stress vs. rest test sessions, (ANOVA repeated measures: AUC of POMS and STAI scores rest-stress x carbohydrate-protein, main effect of stress, p<0.02), indicating that the applied stressor was effective in inducing psychological stress, regardless of the dietary condition. Consumption of the high-protein vs. high-carbohydrate meal did not affect feelings of depression, tension, anger, and anxiety differently (ANOVA repeated measures: change in POMS and STAI scores pre- to post-meal rest-stress x carbohydrate-protein, p>0.1). There were no differences in POMS and STAI scores between men and women, in all conditions.

Simple linear regression models showed that salivary cortisol concentrations were not related to POMS and STAI scores in men and women, in all conditions, when analyzing the AUC, and the change in cortisol concentrations and POMS and STAI scores pre- to post-meal.

### Appetite profile

The fasted state was confirmed by low satiety and fullness scores (11.8±2.5, 9.6±1.9 mmVAS), and high hunger, ‘desire to eat’, and thirst scores (80.6±2.6, 83.9±2.2, 68.1±3.7 mmVAS). Consumption of the lunch resulted in an increase in satiety and fullness scores (Δ = 63.2±4.6, 69.9±3.7 mmVAS, p<0.001), and a decrease in hunger, ‘desire to eat’, and thirst scores (Δ = 67.8±3.3, 68.5±3.3, 33.8±4.3 mmVAS, p<0.001). Conditions of stress vs. rest and of high-protein vs. high-carbohydrate did not affect feelings of hunger, thirst, desire to eat, satiety, and fullness (AUC and per time point).

Consumption of the test meals decreased their subjective liking and wanting (p<0.001; average liking scores pre- and post-meal: 53.5±3.7, 43.4±4.0 mmVAS; average wanting score pre- and post-meal: 65.3±4.3, 8.7±2.0 mmVAS). Conditions of stress vs. rest and of high-protein vs. high-carbohydrate did not influence liking of the test meals pre- and post-meal, confirming that the meals were comparable. The condition of stress vs. rest did not influence wanting of the test-meals pre- and post-meal, though during stress the change in wanting pre- to post-meal was larger in the high-protein condition compared with the high-carbohydrate condition (p = 0.03).

The changes in VAS scores for the appetite profile parameters pre- to post-consumption of the test meals did not differ between men and women.

## Discussion

The main objective of this study was to investigate the possible effects of consumption of meals with different macronutrient contents (high-protein vs. high-carbohydrate) on the physiological cortisol response and on the psychological mood response under stress. Based upon studies of Vicennati et al. [10], Lacroix et al. [11], and Martens et al. [12], we hypothesized that high-protein foods, in contrast to high-carbohydrate foods, would not increase salivary cortisol concentrations more under stress and consequently would improve mood.

In our study the acute psychological stress condition was confirmed by means of POMS and STAI questionnaires, but not endocrinologically by increased salivary cortisol levels. The type of stressor used in the laboratory context might have been too light to elicit a physiological cortisol response [30].

Our study showed a clear meal-induced salivary cortisol response, though no difference in response was detected between consumption of a high-protein lunch and a high-carbohydrate lunch. Some studies have shown that food intake, particularly at lunch, increases cortisol secretion [13], [31], [32], [33]. In contrast, a study by Bray et al. [34] assessing the hormonal responses to a fast-food meal compared with nutritionally comparable meals of different composition, showed no significant salivary cortisol response to meal ingestion. Lovallo et al. [16] showed no meal-induced salivary cortisol response in the case of a mental stressor followed by a meal but did show a meal-induced cortisol response in the case of a physical stressor followed by a meal. This response was higher in women compared with men [16]. The cortisol response to mental stress was smaller in women compared with men [16].

In contrast to our findings, some studies indicated that the macronutrient composition of a meal may influence the magnitude of the cortisol response. Studies by Vicennati et al. [10] and Martens et al. [12] showed higher cortisol levels following a high-carbohydrate meal compared with a high-protein/fat meal. Studies by Gibson et al. [13] and Slag et al. [14] showed increased cortisol levels induced by a protein-rich meal. On the other hand, the study by Bourrilhon et al. [35], investigating the influence of protein- vs. carbohydrate-enriched feedings on physiological responses during an ultra endurance climbing race, showed no effect of diet on serum cortisol levels. It is not clear yet whether the macronutrient composition of a meal can indeed influence cortisol levels. The use of mixed meals instead of single macronutrients, as used in the study by Martens et al. [12], might limit the detection of possible effects of macronutrients on cortisol levels.

Men compared with women participating in our study, showed higher meal-induced salivary cortisol levels and higher overall salivary cortisol levels. According to the review by Kudielka et al. [36], it seems that adult men show higher cortisol responses to psychological stress tasks compared with women, though there are still inconsistencies in literature. Kirschbaum et al. [37] showed sex differences for free salivary cortisol but not for total cortisol stress responses: women taking oral contraceptives and women in the follicular phase had significantly lower free cortisol stress responses than men. In our study there were no differences in salivary cortisol levels between women taking oral contraceptives (n = 11) and women taking no oral contraceptives (n = 8), which is in accordance with studies of e.g. Kirschbaum et al. [37] and Liening et al. [38]. Based on the study of Kirschbaum et al. [37] we hypothesize that the lower salivary cortisol levels in women compared with men might be explained by the fact that women in our study participated during the follicular phase, though the effect seen in the study of Kirschbaum et al. [37] was induced by the psychological stressor, which was not the case in our study. Literature on gender differences concerning meal-induced cortisol increases is scarce.

Men in our study had a larger waist circumference and waist-to-hip ratio compared with women. The meta-regression analysis by de Koning et al. [39] indicated that waist circumference and waist-to-hip ratio are associated with the risk of cardiovascular diseases. It can be hypothesized that the greater cortisol response observed in men may be associated with visceral fat accumulation and an elevated risk for cardiovascular diseases and diabetes and may help explain the higher prevalence for these diseases in men [1], [4], [36], [40].

In contrast to significant gender differences concerning physiological cortisol levels, the psychological mood state did not differ between men and women in our study and physiological cortisol levels were not related to the psychological mood state scores. Moreover, the mood state was not affected by macronutrient composition of the diets. This might be explained by the fact that the high-protein meal and the high-carbohydrate meal were highly comparable, as shown by the VAS scores for the appetite profile parameters. Liking of the test meals and feelings of hunger, thirst, desire to eat, satiety and fullness did not differ between the high-protein and high-carbohydrate condition. It is known from literature that protein is the most satiating macronutrient, and that high-protein meals are more satiating than high-carbohydrate meals [41]. However, our results showed no greater feelings of satiety in the high-protein vs. high-carbohydrate condition. A possible explanation might be that the morning consumption of 50 g of yoghurt was relatively high in protein, and due to this high protein content the lower protein intake and higher carbohydrate intake two hours later might not have resulted in a difference in feelings of satiety at that moment.

In summary, consumption of comparable meals with different macronutrient contents, i.e. high-protein vs. high-carbohydrate, does not influence the physiological cortisol response and the psychological mood response differentially. In our everyday life where stress is a pervasive factor, the development of functional foods, able to regulate the stress response, would be helpful to improve or maintain quality of life, as suggested in the review by Takeda et al. [42]. Foods with the macronutrient contents used in our study seem ineffective in regulating the physiological and psychological stress response. Men in our study showed a higher waist-to-hip ratio and elevated salivary cortisol levels compared with women, which may be associated with an increased risk for cardiovascular diseases and diabetes.

To conclude, consuming a high-protein vs. a high-carbohydrate meal does not influence the physiological cortisol response and the psychological mood response differentially. Men show a higher meal-induced salivary cortisol response compared with women.
